# Efficacy and Tolerance of Thalidomide in Patients With Very Early Onset Inflammatory Bowel Disease

**DOI:** 10.1093/ibd/izad018

**Published:** 2023-02-17

**Authors:** Matteo Bramuzzo, Fabiola Giudici, Serena Arrigo, Paolo Lionetti, Giovanna Zuin, Claudio Romano, Francesco Graziano, Simona Faraci, Patrizia Alvisi, Sara Signa, Luca Scarallo, Stefano Martelossi, Grazia Di Leo

**Affiliations:** Institute for Maternal and Child Health, IRCCS Burlo Garofolo, Trieste, Italy; Bureau de biostatistique et d’épidemiologie, Gustave Roussy and Université Paris-Saclay, Paris, France; Pediatric Gastroenterology and Endoscopy, IRCCS Istituto Giannina Gaslini, Genoa, Italy; Department NEUROFARBA, Meyer Children’s Hospital, University of Florence, Florence, Italy; Pediatrics, Fondazione IRCCS San Gerardo dei Tintori, Monza, Italy; Pediatric Gastroenterology and Cystic Fibrosis Unit, Department of Human Pathology in Adulthood and Childhood G. Barresi, University of Messina, Messina, Italy; Pediatric Unit, Villa Sofia Cervello Hospital, Palermo, Italy; Digestive Endoscopy and Surgery Unit, Bambino Gesù Children’s Hospital, Rome, Italy; Pediatric Gastroenterology Unit, Maggiore Hospital, Bologna, Italy; Pediatric Gastroenterology and Endoscopy, IRCCS Istituto Giannina Gaslini, Genoa, Italy; Department NEUROFARBA, Meyer Children’s Hospital, University of Florence, Florence, Italy; Pediatric Unit, Ca’ Foncello’s Hospital, Treviso, Italy; Institute for Maternal and Child Health, IRCCS Burlo Garofolo, Trieste, Italy

**Keywords:** inflammatory bowel disease, very early onset, thalidomide, therapy, children

## Abstract

**Background:**

Few drugs have been studied for patients with very early onset inflammatory bowel disease (VEOIBD). This study aimed to evaluate the efficacy and tolerance of thalidomide in children with VEOIBD compared with children with pediatric-onset IBD (pIBD).

**Methods:**

A retrospective cohort study with a control group was conducted. Propensity score 1:1 matching was used to identify control subjects. The treatment persistence; the causes of drug withdrawal; the rate of clinical remission and mucosal healing at 1, 2, and 3 years; and adverse events (AEs) were evaluated in children with VEOIBD treated with thalidomide and compared with children with pIBD.

**Results:**

Thirty-nine courses of treatment with thalidomide in VEOIBD and pIBD patients were compared. The treatment persistence at 1, 2, and 3 years was 68.2% (95% confidence interval [CI], 50.8%-80.6%), 57.0% (95% CI, 39.6%-71.1%), and 50.9% (95% CI, 33.7%-65.8%) for VEOIBD patients and 81.7% (95% CI, 65.3%-90.9%), 60.0% (95% CI, 41.7%-74.3%) and 33.0% (95% CI, 17.4%-49.5%) for pIBD patients, respectively (*P* = .12). A significantly higher proportion of VEOIBD patients discontinued therapy due to lack of efficacy (48.2% vs 17.2%; *P* = .03), while AEs were the main reason for discontinuation in pIBD patients. Clinical remission and mucosal healing rates did not significantly differ between VEOIBD and pIBD patients. A significatively lower number of VEOIBD patients experienced AEs compared with pIBD patients (14 [35.9%] vs 30 [76.9%]; *P* = .0005).

**Conclusions:**

Thalidomide is an effective and tolerated treatment in children with VEOIBD. Discontinuation due to lack of efficacy is more frequent, but AEs are less common than in children with pIBD.

**Key Messages**

**What is already known?**

Few drugs have been studied for the treatment of children with very early onset inflammatory bowel disease (VEOIBD), and different responses compared with children with pediatric-onset IBD have been reported.

**What is new here?**

Children with VEOIBD discontinue treatment due to lack of efficacy more frequently but experience fewer adverse events than children with pediatric-onset IBD.

**How can this study help patient care?**

This study offers evidence for the use of thalidomide in children with VEOIBD unresponsive to conventional therapies.

## Introduction

Very early onset inflammatory bowel disease (VEOIBD) refers to IBD, including Crohn’s disease (CD), ulcerative colitis (UC), and IBD unclassified (IBD-U), diagnosed before 6 years of age.^1,2^ VEOIBD comprises a distinct subset of pediatric IBD characterized by stronger genetic predisposition, higher incidence of IBD-U, predominant colonic involvement, more extensive inflammation, more severe course, and peculiar response to treatments.^3^

About 15% of patients with VEOIBD are steroid-refractory, and 30% are steroid-dependent. Moreover, up to 50% of VEOIBD patients require a therapeutic intensification with the use of an anti-tumor necrosis factor α (anti-TNF) biologic within 2 years of diagnosis.^4^

Unfortunately, lower remission rates and higher rates of infliximab discontinuation have been reported in patients with VEOIBD compared with older children, and very few data are available for other biologics.^5,6^ Therefore, there is a need for therapeutic alternatives for children with VEOIBD.

Two randomized controlled trials and several retrospective studies demonstrated that thalidomide, an oral molecule with immunomodulatory, antiangiogenic, and TNF-suppressing properties, is an effective and safe treatment for adults and children with IBD refractory to conventional therapies.^7-9^ However, no data on children with VEOIBD have been published.

The primary aim of this study was to evaluate the efficacy and tolerance of thalidomide in VEOIBD by treatment persistence and reasons for drug withdrawal compared with older children. Secondary aims included the evaluation of clinical and endoscopic remission and adverse event rates.

## Methods

### Population

A multicenter retrospective study with a control group has been conducted involving 8 centers belonging to the Italian Society for Pediatric Gastroenterology, Hepatology and Nutrition, uniformly distributed throughout the Italian territory.

Patients diagnosed with IBD according to the Porto criteria^10^ and treated with thalidomide from June 1, 2003, to June 1, 2022, and with at least 6 months of follow-up were identified within the electronic databases of the participating centers. Patients diagnosed with IBD between 0 and 5 years and treated with thalidomide before 10 years were identified and considered the case group (VEOIBD). Patients with onset and diagnosis of IBD after 10 years of age treated with thalidomide were used as the control group (pediatric-onset IBD [pIBD]). Patients with monogenic forms of IBD were excluded.

Despite concomitant therapies, the disease must have been in a clinical and biochemical active phase at the time thalidomide was started. The decision of starting, modulating, and stopping thalidomide was at the treating physician’s discretion according to the patient’s clinical status.

Demographic and clinical data were collected, including sex, age at diagnosis, IBD type (CD, UC, IBD-U), disease localization, and behavior according to the Paris classification,^11^ associated autoimmune diseases and prior therapies. Details on thalidomide treatment included age at thalidomide start, initial dose, concomitant therapies, duration of treatment, and cause of suspension. Adverse events (AEs) during treatment were also collected. In the case of patients with multiple treatment courses with thalidomide, all courses were included and considered independent if separated by at least 6 months.

To assess the clinical and biochemical efficacy of thalidomide treatment, the Pediatric Crohn’s Disease Activity Index for CD and Pediatric Ulcerative Colitis Activity Index for UC and IBD-U, erythrocyte sedimentation rate (ESR), C-reactive protein (CRP), and fecal calprotectin (FC) the time of thalidomide start and after 3, 6, 12, 24, and 36 months were recorded. Weight and height were registered at baseline, 12 months, and the end of follow-up and expressed as *z* scores according to the Centers for Disease Control and Prevention and World Health Organization’s growth charts.

The grade of endoscopic activity was determined according to the Simple Endoscopic Score for Crohn’s Disease and Mayo score for CD and UC/IBD-U, respectively, at the times mentioned previously.

### Outcome Measures

Treatment persistence was defined as the time between the start and the last dose of thalidomide. Clinical remission was defined by a Pediatric Crohn’s Disease Activity Index or Pediatric Ulcerative Colitis Activity Index <10. ESR, CRP, and FC were considered normal when <20 mm/h, < 0.5 mg/dL, and <100 mg/kg, respectively.

Mucosal healing was defined as a score of <2 in the Simple Endoscopic Score for Crohn’s Disease or 0 in the Mayo score. FC levels <100 mg/kg were used as a surrogate marker of mucosal healing in patients who did not undergo an endoscopic evaluation.

Prolonged remission was defined by at least 12 months of clinical, biochemical, and endoscopic remission.

### Statistical Analysis

All statistical analyses were performed using R software (version 4.0.2; R Foundation for Statistical Computing).

The 2-sided *z* test with unpooled variance was used to estimate the sample size. Considering the persistence of treatment as the main outcome, it was estimated that 50% of patients with VEOIBD and 20% of patients with pIBD would achieve the 3-year term of therapy. Setting the beta at 20% and alpha at 0.05%, it was calculated that at least 36 VEOIBD and 36 pIBD patients needed included to have sufficient power (80%) to detect a difference between the group proportions of 30%.

Propensity score matching (1:1) was performed to balance the selection bias and potential confounding factors between VEOIBD and pIBD patients. Propensity score matching was calculated based on baseline characteristics, including age, sex, and type of IBD. The R package MatchIt (https://kosukeimai.github.io/MatchIt/) (1:1 matching with the nearest neighbor) was used for the propensity matching process for IBD group selection.

The Mann-Whitney *U* test was used to compare continuous data between VEOIBD patients and patients with pIBD. The chi-square test and Fisher exact test were used for categorical variables.

The Kaplan-Meier method was used to estimate treatment persistence at different follow-up times. The log-rank test was performed to compare persistence rates between VEOIBD and pIBD groups. The cumulative incidence function, which considers competing risks,^12^ was used to describe the reasons for treatment interruption. Then, the cumulative incidence of relapse in the VEOIBD and pIBD groups was compared with the Gray test. Cumulative incidence function and competing risk analyses were performed with the R package cmprsk.^13^ Univariate logistic regression models were performed to investigate potential predictive factors of treatment interruption before 1 year for all cohorts and stratifying for VEOIBD and pIBD groups. Results were reported using odds ratio and 95% confidence interval (CI).

Continuous data are presented as median (interquartile range [IQR]) and categorical data are presented as absolute number and percentage.

All statistical tests were 2-tailed. A *P* value <.05 was considered significant.

## Results

### Population

Thirty-nine VEOIBD patients were identified; all were screened with a clinical, immunological, or genetic (targeted next-generation sequencing panels or whole exome sequencing) workup for monogenic forms of IBD. Two patients were excluded because of the diagnosis of chronic granulomatous disease and Loeys-Dietz syndrome. Two patients received 2 courses of therapy with thalidomide at an interval of 2 years. Thus, 39 courses of treatment in VEOIBD patients were considered. Thalidomide was started before 6 years of age in 22 VEOIBD patients and between 6 and 9 years of age in 17 VEOIBD patients.

Forty-five patients with pIBD were identified and their data were collected. After the application of the propensity score, 39 courses of thalidomide from 39 pIBD patients were included.

Baseline characteristics of patients are presented in Table 1. No patient with pIBD had IBD-U. Children with very early onset CD had a predominance of colonic localization, while ileocolonic involvement was more common in patients with pIBD. Most VEOIBD and pIBD patients had refractory disease and were resistant to multiple treatments. Eight (20.5%) VEOIBD patients and 10 (25.6%) pIBD patients were not exposed to biologics.

**Table 1.** Patient characteristics

	VEOIBD (n = 39)	pIBD (n = 39)	*P* Value
Sex			
Female	13 (33.3)	14 (35.9)	.81
Male	26 (66.7)	25 (64.1)	
IBD type			
Crohn’s disease	14 (35.9)	16 (41.0)	.64
Ulcerative colitis	20 (51.3)	23 (59.0)	.49
IBD-U	5 (12.8)	0	.05
Age at diagnosis, y	2.4 (1.4 to 4.6)	12.3 (10.5 to 13.6)	<.01
Age at thalidomide, y	5.5 (3.5 to 7.2)	14.4 (13.2 to 15.7)	<.01
Duration of disease, y	2.2 (1.0 to 3.2)	2.1 (1.0 to 3.0)	.47
Disease activity			
PCDAI	29.7 (20.0 to 35.0)	35.0 (22.5 to 44.4)	.80
PUCAI	41.6 (30.0 to 50.0)	43.0 (32.5 to 57.5)	.83
Weight *z* score	-0.51 (-1.40 to 0.01)	-0.43 (-1.35 to 0.37)	.70
Height *z* score	-0.85 (-1.42 to 0.08)	-0.17 (-0.84 to 0.24)	.98
Associated diseases			
Autoimmune cholangitis	2 (5.1)	1 (2.6)	1.00
Celiac disease	1 (2.6)	1 (2.6)	1.00
Vitamin D–dependent rickets	1 (2.6)	0 (2.6)	1.00
IgA nephropathy	1 (2.6)	0 (2.6)	1.00
Localization			
Crohn’s disease^a^			
L1	2 (14.3)	3 (18.7)	1.00
L2	9 (64.3)	0 (0.0)	<.01
L3	3 (21.4)	13 (81.3)	<.01
L4a	1 (7.1)	5 (31.2)	.17
L4b	2 (14.3)	5 (31.2)	.40
Ulcerative colitis and IBD-U^b^			
E1	0	1 (4.3)	.48
E2	0	1 (4.3)	.48
E3	2 (8.0)	2 (8.7)	1.00
E4	23 (92.0)	19 (82.6)	.41
Behavior			
Crohn’s disease^c^			
B1	12 (85.7)	9 (56.3)	.12
B2	1 (7.1)	6 (37.5)	.09
B3	1 (7.1)	1 (6.3)	1.00
B2B3	0	0	1.00
p	4 (28.6)	10 (62.5)	.08
Ulcerative colitis and IBD-U^d^			
S0	10 (40.0)	7 (30.4)	.55
S1	15 (60.0)	16 (69.6)	.55
Previous treatment			
Steroids	38 (97.4)	35 (89.7)	.35
Exclusive enteral nutrition	8 (20.5)	7 (17.9)	1.00
Aminosalicylates	23 (59.0)	22 (56.4)	1.00
Azathioprine	30 (76.9)	27 (69.2)	.61
Methotrexate	6 (15.4)	3 (7.7)	.48
Infliximab	28 (71.8)	27 (69.2)	.80
Adalimumab	9 (23.1)	11 (28.2)	.79
Ustekinumab	0	1 (2.6)	1.00
Vedolizumab	0	1 (2.6)	1.00
Cyclosporin A	3 (7.7)	1 (2.6)	.61
Thalidomide	2 (5.1)	0	.49
1 biologic	31 (79.5)	29 (74.4)	.79
2 biologics	6 (15.4)	13 (33.3)	.11
3 biologics	0	1 (2.6)	1.00
Surgery	3 (7.7)	1 (2.6)	.61
Concomitant treatment			
Steroids	23 (59.0)	28 (71.8)	.23
Partial enteral nutrition	1 (2.6)	3 (7.7)	.62
Aminosalicylates	10 (25.6)	4 (10.6)	.14
Azathioprine	2 (5.1)	1 (2.6)	1.00
Methotrexate	3 (7.7)	0	.24
Infliximab	0	0	1.00
Adalimumab	1 (2.6)	0	1.00

Values are n (%) or median (interquartile range).

Abbreviations: IBD, inflammatory bowel disease IBD-U, inflammatory bowel disease unclassified; IgA, immunoglobulin A; PCDAI, Pediatric Crohn’s Disease Activity Index; pIBD, pediatric-onset inflammatory bowel disease; PUCAI, Pediatric Ulcerative Colitis Activity Index; VEOIBD, very early onset inflammatory bowel disease

^a^L1: distal 1/3 ileum ± limited cecal disease; L2: colonic disease; L3: ileocolonic disease; L4a: upper disease proximal to ligament of Treitz; L4b: upper disease distal to the ligament of Treitz and proximal to the distal 1/3 ileum; L4ab: upper disease involvement in both L4a and L4b.

^b^E1: ulcerative proctitis; E2: left-sided UC (distal to splenic flexure); E3: extensive (hepatic flexure distally); E4: pancolitis (proximal to hepatic flexure).

^c^B1: nonstricturing nonpenetrating behavior; B2: stricturing behavior; B3: penetrating behavior; B2B3: both B2 and B3.

^d^S0: never severe (PUCAI <65); S1: ever severe (PUCAI ≥65).

The 2 groups had no significant differences in clinical scores and biochemical parameters at baseline.

Weight and height *z* scores were similar between VEOIBD and pIBD patients (-0.51 [IQR, -1.40 to 0.01] vs -0.43 [IQR, -1.35 to 0.37]; *P* = .70; and -0.85 [IQR, -1.42 to 0.08] vs -0.17 [IQR, -0.84 to 0.24]; *P* = .98, respectively).

Concomitant treatments, mainly steroids and aminosalicylates, and the starting dose of thalidomide was similar between VEOIBD and pIBD patients (2.0 [IQR, 1.7 to 2.2] mg/kg and 1.9 [IQR, 1.6 to 2.1] mg/kg, respectively; *P* = .25).

### Drug Persistence

At the study retrieval date, 12 (30.8%) VEOIBD and 10 (25.6%) pIBD patients were still on thalidomide after 3.6 (IQR, 2.3 to 4.1) and 1.3 (IQR, 0.9 to 2.4) years of treatment. Twenty-seven (69.2%) VEOIBD and 29 (74.4%) pIBD patients had stopped thalidomide (*P* = .61) after 1.5 (IQR, 0.5 to 4.7) and 1.9 (IQR, 0.8 to 2.7) years of treatment, respectively (*P* = .95). The maximum treatment duration was 9.7 years for VEOIBD and 5.8 years for pIBD patients.

**Figure 1.**
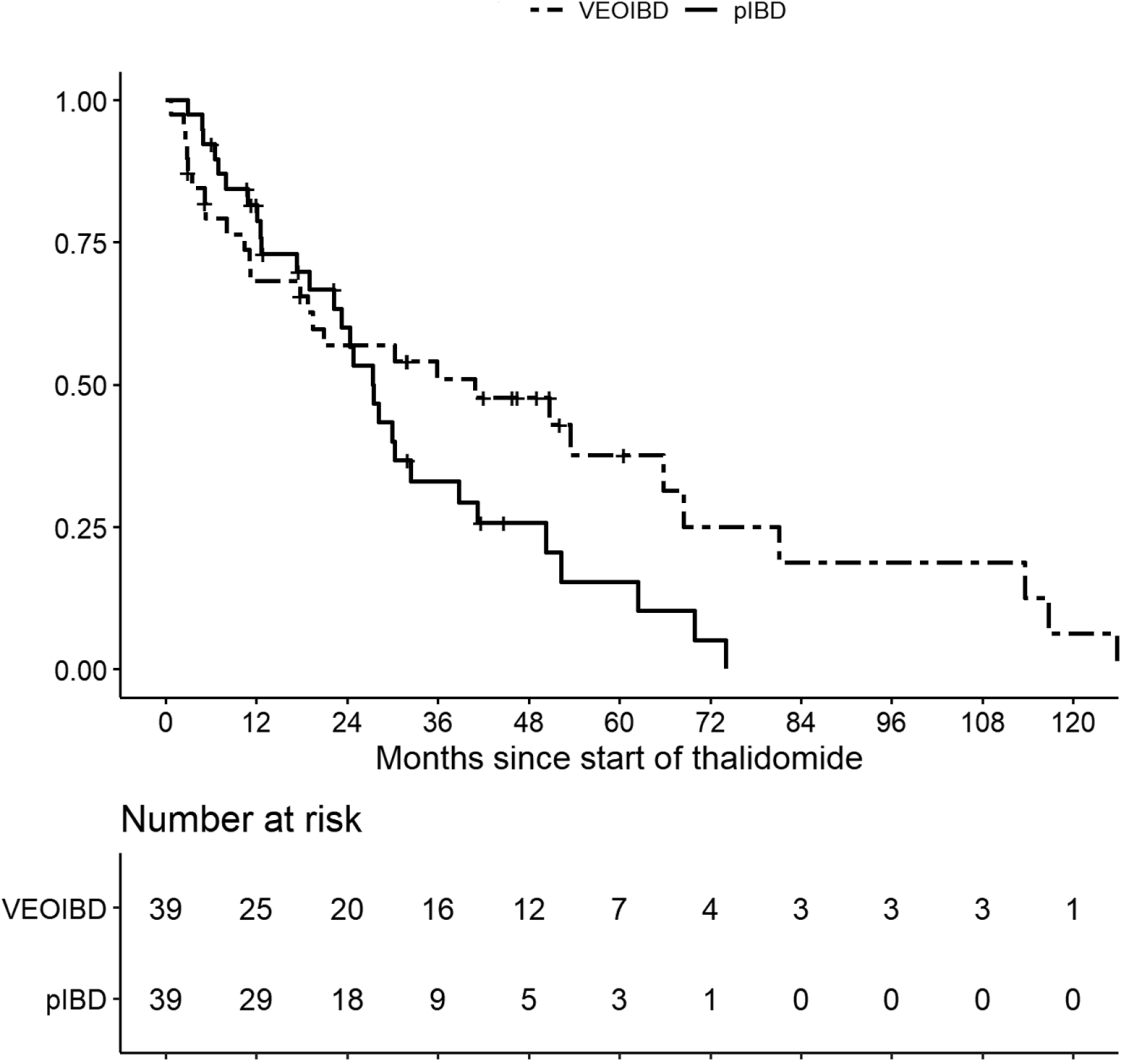
Persistence to thalidomide according to the 2 groups of patients. pIBD, pediatric-onset inflammatory bowel disease; VEOIBD, very early onset inflammatory bowel disease.

Considering all possible reasons for discontinuing the thalidomide treatment, the persistence rates at 1, 2, and 3 years were 68.2% (95% CI, 50.8%-80.6%), 57.0% (95% CI, 39.6%-71.1%), and 50.9% (95% CI, 33.7%-65.8%) for the VEOIBD group and 81.7% (95% CI, 65.3%-90.9%), 60.0% (95% CI, 41.7%-74.3%), and 33.0% (95% CI, 17.4%-49.5%) for the pIBD group, respectively (*P* = .10) (Figure 1).

A significantly higher proportion of VEOIBD patients discontinued therapy due to lack of efficacy or relapse (48.2% vs 17.2%; *P* = .03). In contrast, AEs were the leading cause of discontinuation in pIBD patients. Two (7.4%) VEOIBD and 7 (24.1%) pIBD patients stopped treatment after having achieved a prolonged remission on thalidomide following a therapeutic de-escalation strategy (Table 2).

**Table 2.** Reasons of thalidomide withdrawal

Reason	VEOIBD (n = 27)	pIBD (n = 29)	*P* Value
Adverse events	10 (37.0)	16 (55.2)	.28
Poor compliance	2 (7.4)	1 (3.5)	.95
Relapse	13 (48.2)	5 (17.2)	.03
Prolonged remission	2 (7.4)	7 (24.1)	.18

Values are n (%).

Abbreviations: pIBD, pediatric-onset inflammatory bowel disease; VEOIBD, very early onset inflammatory bowel disease.

**Figure 2.**
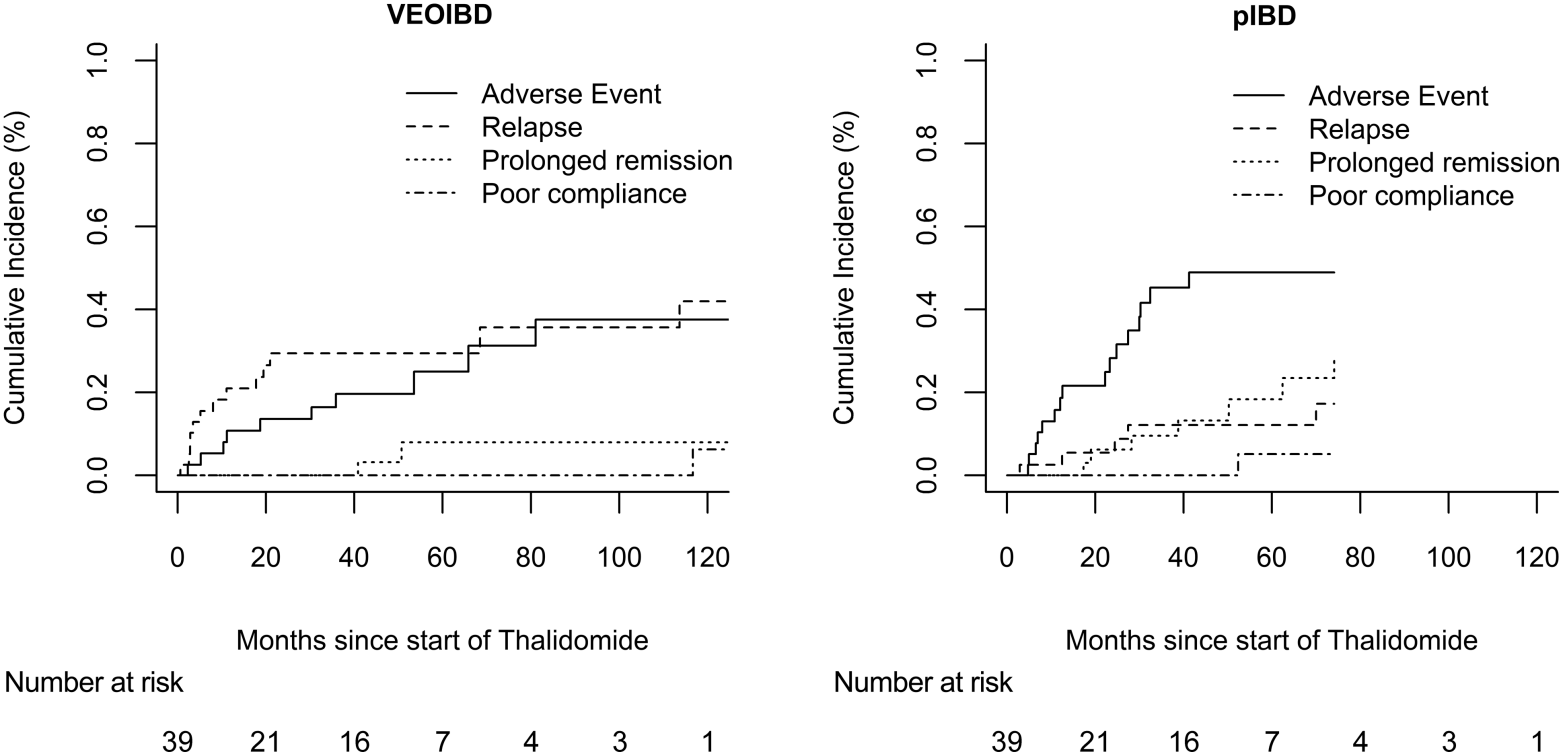
Cumulative incidence of all reasons for withdrawal from treatment. pIBD, pediatric-onset inflammatory bowel disease; VEOIBD, very early onset inflammatory bowel disease.

Figure 2 shows the cumulative incidence of all reasons for treatment stop.

The swimmer plot in Supplementary Figure 1 shows a comprehensive view of each patient’s treatment persistence over time and reason for treatment stop.

### Efficacy

Twenty-four (63.2%) of 38, 22 (78.6%) of 28, 21 (84.0%) of 25, 17 (85.0%) of 20, 15 (88.2%) of 17 patients with VEOIBD were in remission at 3, 6, 12, 24, and 36 months, respectively. The remainder of the patients who reached the assessment time point and who were not in remission had mild disease activity.

Mucosal healing was achieved by 4 (21%) of 19, 7 (37%) of 19, 5 (31%) of 16, 9 (64%) of 14, 6 (60%) of10 patients with VEOIBD at 3, 6, 12, 24, and 36 months, respectively.

No significant difference was found in evaluating efficacy according to IBD type or to the prior exposure to biologics.

**Figure 3.**
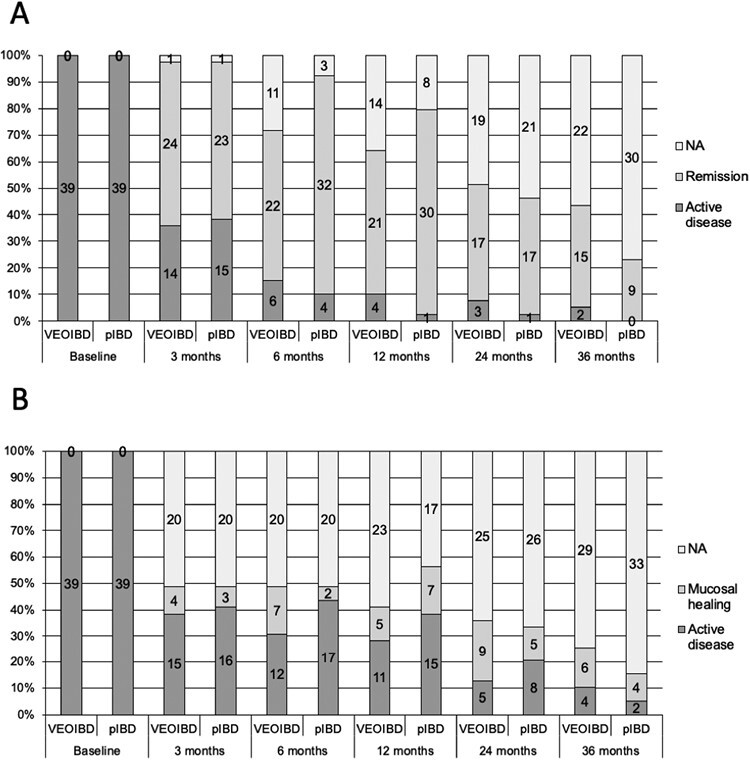
Clinical remission and mucosal healing rates in children with very early onset inflammatory bowel disease (VEOIBD) and pediatric-onset IBD (pIBD). A, Clinical remission; B, mucosal healing. Not available (NA) refers to patients for whom the clinical and endoscopic outcome cannot be assessed due to the failure to reach the time point or the missed evaluation mucosal healing (unavailability of endoscopy or fecal calprotectin).

When directly compared, remission and mucosal healing rates were not significantly different between VEOIBD and pIBD patients (Figure 3). However, the analysis based on the competitive risks showed that at 12 months, there was a trend toward a higher incidence of remission in pIBD patients (96.8% vs 85.2%; *P* = .12)

**Figure 4.**
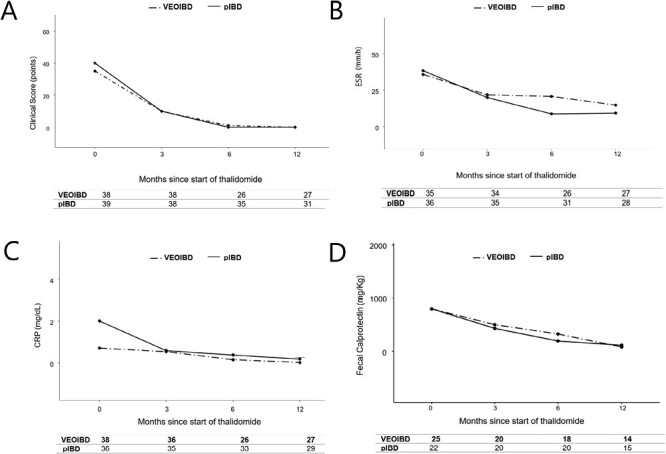
Trends of clinical and biochemical parameters over time in the 2 groups of patients. A, Clinical scores; B, erythrocyte sedimentation rate (ESR); C, C-reactive protein (CRP); D: fecal calprotectin. Results are reported using medians. pIBD, pediatric-onset inflammatory bowel disease; VEOIBD, very early onset inflammatory bowel disease.

In the first year of treatment, the medians of the clinical scores were identical and CRP and FC levels decreased with a similar trend in the 2 groups. Median ESR levels were significantly higher in VEOIBD children at 6 (*P* = .007) and 12 (*P* = .011) months than in pIBD children (Figure 4).

Weight and height *z* scores had a similar improvement between VEOIBD and pIBD patients after 1 year of treatment (-0.22 [IQR, -0.54 to 0.73] vs 0.21 [IQR, -0.23 to 1.00]; *P* = .87; and -0.24 [IQR, -1.14 to 0.42] vs -0.10 [IQR, -0.80 to 0.40]; *P* = .68, respectively) and at the end of the follow-up (-0.06 [IQR, -0.98 to 0.75] vs 0.13 [IQR, -0.58 to 0.77]; *P* = .97; and -0.28 [IQR, -1.00 to 0.27] vs 0.00 [IQR, -0.58 to 0.33]; *P* = .97, respectively).

The thalidomide dose was decreased in both groups. Among patients who stopped treatment, the thalidomide dose was lower in the pIBD group than in the VEOIBD group (0.9 [IQR, 0.4 to 1.6] mg/kg/d vs 1.3 [IQR, 0.9 to 1.8] mg/kg/d; *P* = .04).

Steroids were suspended in all patients except for 1 pIBD patient who stopped thalidomide after 1 month; other concomitant treatments were discontinued during thalidomide treatment in most patients (28 [71.8%] VEOIBD and 29 [74.4%] IBD patients; *P* = 1.00). Six VEOIBD and 4 pIBD patients continued mesalamine; 1 VEOIBD and 1 pIBD patient continued azathioprine.

### Tolerance

A significantly lower number of patients with VEOIBD experienced AEs compared with pIBD patients (14 [35.9%] vs 30 [76.9%]; *P* = .0005).

AEs caused treatment discontinuation in 8 (57.1%) and 14 (46.6%) VEOIBD and pIBD patients, respectively (*P* = .77). The type of AE is reported in Table 3. All patients who had central neuropathy, amenorrhea, leukopenia, or fatigue had a complete recovery. Eighteen patients (9 VEOIBD, 9 pIBD) resolved peripheral neuropathy, 7 patients (2 VEOIBD, 5 pIBD) had clinical improvement with persistence of electromyographic alterations, and 11 patients had an unknown outcome at follow-up.

**Table 3.** Adverse events

Adverse event type	VEOIBD (n = 14)	pIBD (n = 30)	*P* Value
Central neuropathy	3 (21.4)	5 (16.7)	.69
Vertigo	1 (7.1)	0	.32
Pseudotumor cerebri	2 (14.3)	2 (6.7)	.58
Transitory ischemic attack	0	3 (10.0)	.54
Peripheral neuropathy	12 (85.7)	23 (86.7)	.69
Amenorrhea	0	2 (6.7)	1.00
General weakness	0	2 (6.7)	1.00
Leukopenia	0	1 (3.3)	1.00
Total adverse events	15	36	

Values are n (%).

Abbreviations: pIBD, pediatric-onset inflammatory bowel disease; VEOIBD, very early onset inflammatory bowel disease.

### Factors Related to Treatment Persistence

No clinical or biochemical factor predictive of treatment persistence and remission at 12 months was identified.

## Discussion

Most of the drugs used in IBD patients are off-label under 6 years of age, and, due to the unavailability of evidence on the efficacy and safety of therapies, children with VEOIBD are at risk of not receiving appropriate treatment. Rediscovering old and often neglected drugs can lead to an effective treatment that avoids the long-term consequences of uncontrolled inflammation.

This study demonstrates that thalidomide can be effective and tolerated by children with VEOIBD refractory to standard treatments. Indeed, about half of the VEOIBD patients achieved clinical remission, and a third of those who continued thalidomide and had FC or endoscopy performed achieved mucosal healing within 1 year of treatment. Moreover, weight and height growth also improved while patients were on thalidomide.

This finding appears significant for many reasons. First, the study population had a severe disease: almost all children were unresponsive to conventional therapies and had already failed at least 1 biological drug. Second, persistence to therapy and remission rates were higher than reported for other drugs. Indeed, 2 studies showed that only 19% to 36% of infliximab-treated children with VEOIBD were still on treatment at 12 months of therapy; moreover, the response rate at 12 months was 10% to 11% in patients with CD, 7% to 25% in patients with UC, and 0% in patients with IBD-U.^6,14^ Only recently, a North American study reported higher rates of anti-TNF persistence, likely due to differences in standard anti-TNF dosing or concomitant use of immunomodulators.^15^

Vedolizumab has been studied in 16 patients with VEOIBD. The drug was started at a median age of 6.5 years, although some began after 10 years of age. At the fourth dose week (end of the induction phase), clinical response was observed in 56.3% of patients, while remission was reported in 37.5% of patients (2 CD and 4 UC).^16^

Ustekinumab has not been systematically evaluated in children with VEOIBD, but in a Japanese case series, 3 VEOIBD patients were included, and 1 achieved remission.^17^ Although a direct comparison cannot be made, it is possible that the higher efficacy and persistence observed for thalidomide vs conventional therapies and biologics lies in its multiple and nonselective mechanisms of action including the inhibition of TNF, interferon γ, and interleukin-12; integrin expression; and angiogenesis.^18^

As reported for infliximab,^6^ the rates of thalidomide discontinuation due to ineffectiveness or relapse were higher in VEOIBD patients than in adolescents, at least in the first year of treatment. It has been shown for drugs such as azathioprine and infliximab that age-related differences in metabolism can affect drug efficacy, and higher doses are required in patients with VEOIBD to achieve the same therapeutic effect.^5,6,19^ This study could not investigate whether there are any pharmacological differences between VEOIBD and adolescents causing the higher rates of thalidomide failure in the first months of treatment in children with VEOIBD. However, it seems more likely that the differences in drug response may lie in the intrinsic characteristics of the disease and a more severe phenotype. as suggested by the higher ESR values during follow-up.

Although fewer VEOIBD patients benefit from the drug in the first 2 years of treatment, the long-term persistence was higher. In particular, a group of patients responding to treatment remained in stable remission for very long periods. Notably, remission was maintained despite the suspension of concomitant treatments, mainly steroids, and the reduction of thalidomide dose. The latter is a safety practice that has been shown to limit the cumulative dose, considered a risk factor for developing adverse effects.^20^ Furthermore, 2 VEOIBD patients discontinued thalidomide after a long period of remission and received a second course of the drug following a relapse unresponsive to other therapeutic lines. The possibility of a drug holiday with a complete response after retreatment is an intriguing strategy to reduce the risk of AEs related to treatment^21^ and broadens the possibilities of using thalidomide in selected cases.

While a high incidence of AEs has been reported for infliximab-treated children with VEOIBD,^6^ AEs were uncommon in patients with VEOIBD treated with thalidomide. Moreover, they led to therapy discontinuation less frequently than in adolescents. However, the safety profile remains a sensitive point in the use of thalidomide because serious adverse effects also occurred in VEOIBD patients and involved mainly the peripheral and central nervous systems. Fortunately, all serious AEs were recovered and only few patients continued to have signs of mild peripheral neuropathy. The risk of teratogenicity and secondary amenorrhea was not possible in girls with VEOIBD who were prepubertal, but whether thalidomide affected ovarian reserve^22^ and the development of primary amenorrhea cannot be evaluated.

This study has some limitations that should be acknowledged, starting from its retrospective design and the low number of patients, which do not give us enough power to draw definitive conclusions on the secondary outcomes. However, VEOIBD are relatively rare diseases, and this is one of the largest cohorts ever presented with a very long follow-up.

Moreover, the comparison between VEOIBD and adolescents is complicated by the basal differences that diseases have in the 2 populations, as demonstrated by the distribution of the number of IBD-U. We have attempted to overcome this problem through a rigorous statistical coupling that considered the patient’s main clinical and demographical characteristics. However, dealing with a small number of patients, substantial imbalances of some covariates (ie, disease location) were unavoidable despite using a sensibly estimated propensity score.

Finally, the inclusion of 2 therapeutic cycles for 2 VEOIBD patients, although separated by a long time interval, may have introduced a bias in the evaluation of drug efficacy. However, this inclusion however allows a broad and complete description of a real-life experience. Similarly, prolonged remission was considered among the causes of treatment discontinuation because this reflected what was done in the real life. To avoid biases, the causes of treatment discontinuation were evaluated with a competitive risk analysis.

Despite these limitations, this is the first study that provides extensive evidence of the effect of thalidomide in children with VEOIBD and comparison with older patients. These findings allow considering the use of thalidomide in children with VEOIBD refractory to conventional or biological therapies or unsuitable for anti-TNF biologics.

## Conclusions

Thalidomide is an effective and tolerated treatment in children with VEOIBD. Lack of efficacy causes treatment discontinuation more frequently than in children with pIBD. Still, in VEOIBD patients who respond, maintenance can be sustained for very long periods. Moreover, AEs are less common than in pIBD patients but may still be severe. Larger studies are needed to confirm these findings and to establish which children with VEOIBD can benefit most from thalidomide therapy and which sequence of use in relation to the new biologics is desirable.

## Supplementary Material

izad018_suppl_Supplementary_Figure_1
