# Supplementary figures and images for: Efficacy and Tolerance of Thalidomide in Patients With Very Early Onset Inflammatory Bowel Disease
Source: Inflamm Bowel Dis. 2023 Feb 17;30(1):20–8. doi: 10.1093/ibd/izad018 (PMC10769807; doi:10.1093/ibd/izad018)

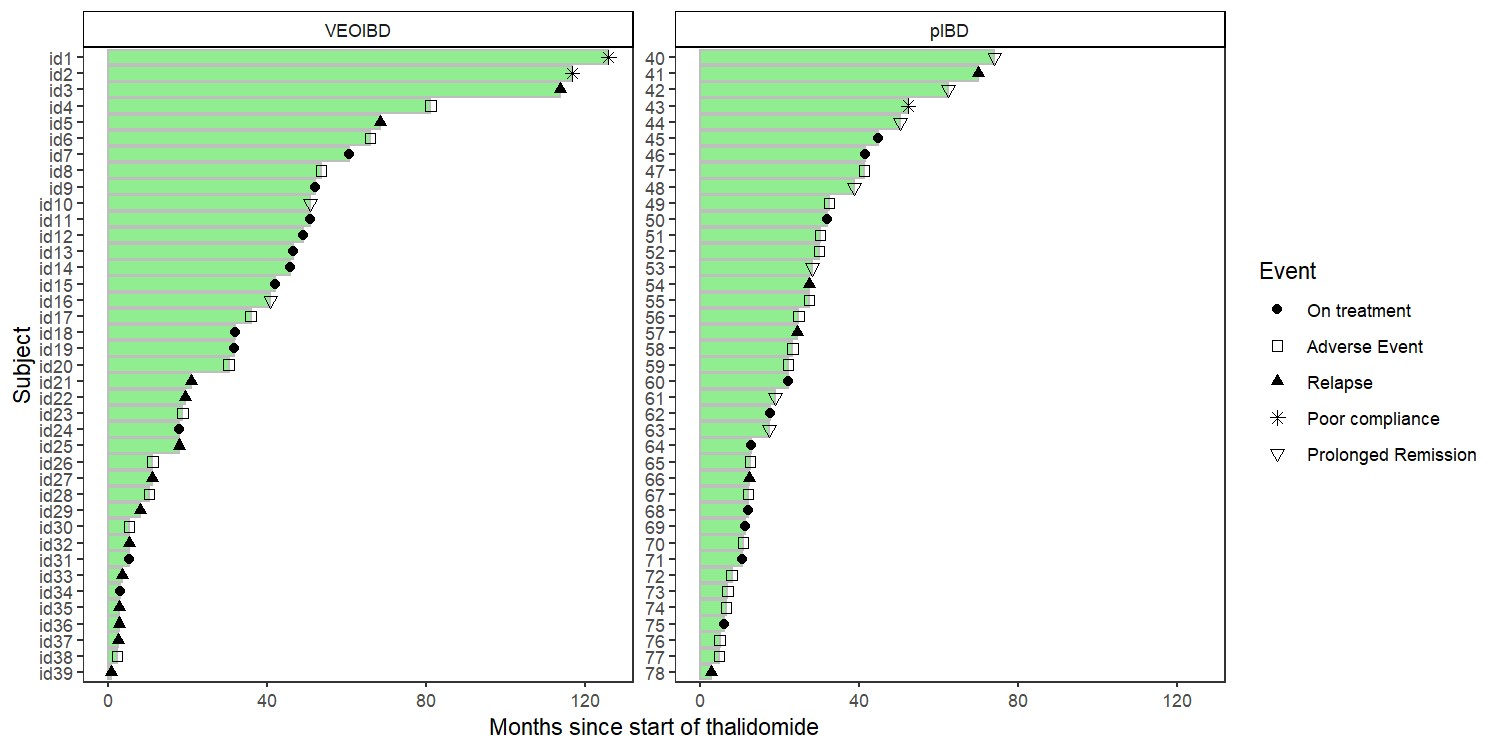

Supplement: izad018_suppl_Supplementary_Figure_1 [file izad018_suppl_supplementary_figure_1.jpeg]
